# Prepatellar Bursitis with Abscess due to *Corynebacterium ulcerans*

**DOI:** 10.1155/2021/3507672

**Published:** 2021-07-27

**Authors:** Sachin M. Patil, Phillip Paul Beck, Taylor B. Nelson, Andres Bran Acevedo, William Roland

**Affiliations:** ^1^Department of Medicine, Division of Pulmonary, Critical Care and Environmental Medicine, University of Missouri Hospital and Clinic, One Hospital Dr, Columbia, MO 65212, USA; ^2^Department of Medicine, Division of Infectious Disease, University of Missouri Hospital and Clinic, One Hospital Dr, Columbia, MO 65212, USA

## Abstract

*Corynebacteria* are ubiquitous and reside as skin and mucosa commensals in animals. They are considered contaminants in clinical specimens, but significant clinical data points to their virulence and pathogenic potential over the last two decades. *Corynebacteria* can cause both community-acquired and nosocomial infections. *Corynebacterium diphtheriae* (*C. diphtheriae*) responsible for diphtheria has declined over the previous two decades with an increase in a similar clinical syndrome by *Corynebacterium ulcerans* (*C. ulcerans*) in Europe. As per recent studies, *C. ulcerans* shares similar virulence factors with *C. diphtheriae*. *C. ulcerans* has been implicated in airway infections, skin and soft tissue infections, lymphadenitis, wound infections, and rarely necrotizing fasciitis. Pet or farm animals have been the source of these infections to humans, as per recent reports. Strains can be either toxigenic or nontoxigenic. Due to recent advances, methods to characterize strains have become easier with mass spectrometry. Antimicrobial susceptibility testing is a must for definite treatment as *C. ulcerans* can be resistant to first-line antibiotic therapy. If resources are available, it is prudent to find if there is any toxin production. Here, we describe a rural farmer in central Missouri presenting with acute-onset right knee pain diagnosed with right prepatellar bursitis with abscess due to *C. ulcerans* infection. He recovered with surgical debridement and antimicrobial therapy. This is the first case of *C. ulcerans* causing prepatellar bursitis with an abscess as per medical literature review.

## 1. Introduction

*Corynebacterium* is an aerobic nonsporulating gram-positive rod [1]. *Corynebacterium diphtheriae* (*C. diphtheriae*) is a well-known subspecies of this group, which causes diphtheria, a potentially lethal disease in the pediatric population and unvaccinated adults associated with significant morbidity and long-term sequelae. Diphtheria is a public health issue in countries with inadequate vaccination strategies. Incidence in the United States of America (USA) is <0.01 case per 100,000 persons [2]. Two other subspecies that produce a virulent factor toxin are *Corynebacterium pseudotuberculosis* (*C. pseudotuberculosis*) and *Corynebacterium ulcerans* (*C. ulcerans*) [3, 4]. Both produce disease in livestock such as cattle, goats, and sheep and pet animals (cats or dogs). Infected livestock and animal pets serve as reservoirs for this infection. Zoonotic transmission occurs via raw contaminated milk consumption or contact with wound discharge or animal secretions [3]. In some European countries, it is the leading cause of cutaneous diphtheria in adults [4, 5]. Respiratory diphtheria due to *C. ulcerans*, mostly from industrialized countries, has increased over the last few decades as well [3]. In the USA, from 1996 to 2018, *C. ulcerans* was responsible for causing airway disease in five patients (culture positive). All five individuals' vaccination status against diphtheria was inadequate [2]. A single conjunctivitis case by a toxigenic strain was reported from Missouri in 2018, possibly from a pet animal exposure [6]. Animal exposure is not the source for all human cases of *C. ulcerans*, although human-to-human transmission has not been proven yet [3, 4]. A PubMed literature search revealed no prior case reports of *C. ulcerans* as a cause of prepatellar bursitis with abscess. Here, we report the case of a rural hog farmer who presented with acute-onset right knee pain diagnosed with right prepatellar bursitis with abscess due to *C. ulcerans* infection.

## 2. Case Presentation

**Figure 1:**
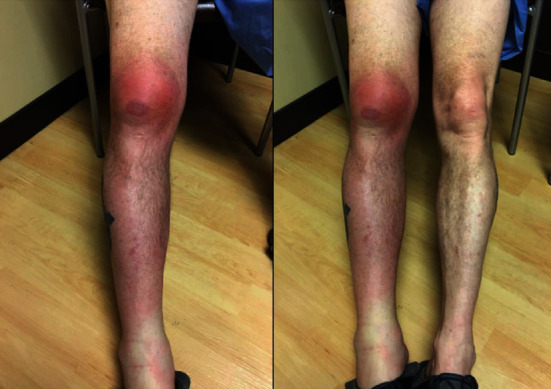
Right leg prepatellar bursitis with extension of inflammation to the upper medial thigh and lower leg.

**Figure 2:**
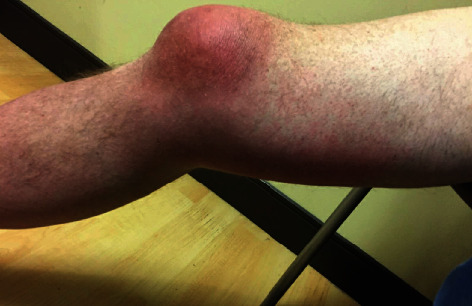
Lateral view of the right knee joint revealing right knee prepatellar bursitis with extension of inflammation.

A 53-year-old male patient presented to the orthopedic clinic at our institution for new-onset right knee pain of two-day duration. His past medical history was significant for type two diabetes mellitus, acid reflux disorder, chronic obstructive pulmonary disease, and left shoulder osteoarthritis. His smoking history consisted of 1.5 packs per day with 40 pack-years with no alcohol or substance abuse. He is a self-employed hog farmer. One day before the presentation, he noticed progressive soft tissue swelling over the right knee with erythema extending from the midthigh down to below the knee. The right knee pain was described as intense and sharp with a grade of 10/10 with radiation to the right hip and difficulty in right lower extremity weight-bearing. There were no associated fevers, chills, night sweats, nausea, vomiting, or change in left shoulder pain. There was no history of right knee trauma, gout, or intra-articular steroid injection. His job demanded him to be on his knees during the day. A general observation revealed a middle-aged male in mild distress. Clinical examination showed stable vital signs and an antalgic gait. The direct clinical inspection was notable for right lower extremity diffuse swelling from the midthigh to the foot along with significant erythema (Figures 1 and 2). Palpation revealed tenderness over the superior portion of the patella with no effusion. His knee flexion was limited to 100 degrees with no joint laxity. The right knee X-ray displayed prepatellar soft tissue swelling without osseous abnormality. A transfer to the emergency department (ED) for further care was initiated.

**Figure 3:**
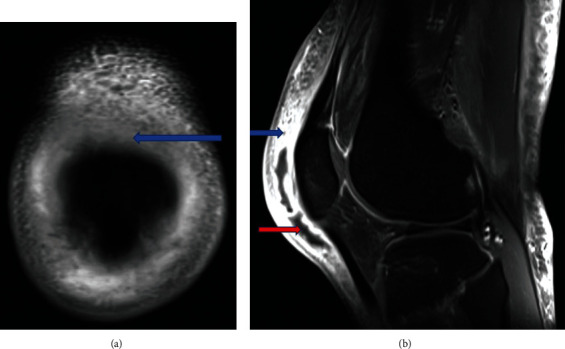
(a) MRI coronal view reveals an area of hypodensity in the prepatellar bursa indicating bursitis (blue arrow). (b) Sagittal T1 view reveals prepatellar bursitis (blue arrow) and abscess (red arrow).

At the ED, the patient was tachycardic with stable blood pressure and oxygenation. Labs revealed a leukocytosis of 13,250/mL with an erythrocyte sedimentation rate of 76 mm/hr (0–40 mm/hr), C reactive protein of 6.74 mg/dL (<0.4 mg/dL), and a normal metabolic panel. Hemoglobin A1c was 7.4%, indicative of well-controlled diabetes mellitus. Right knee ultrasound showed generalized knee and calf subcutaneous edema. A 1.7 × 1.6 × 0.6 cm fluid collection along the deep subcutaneous tissue at the knee joint level was present along with the anterior medial knee with no knee joint involvement. Two sets of blood cultures were sent. The patient received Toradol for pain and intravenous (IV) clindamycin empirically. A bedside abscess aspiration was futile, and he was admitted to the internal medicine (IM). Orthopedic surgery recommended right knee magnetic resonance imaging (MRI). Due to suspected infection, IV clindamycin was stopped, and vancomycin was started. The next day, right knee MRI displayed a prepatellar bursa abscess (1 × 13.5 × 6 cm) with surrounding bursitis and myositis (Figures 3(a) and 3(b)). Preoperatively, he was given a single IV dose of cefazolin 2 gm (Gram) and dexamethasone 8 mg. An incision and drainage of the prepatellar bursitis revealed a small amount of murky fluid; it was sent for gram stain in addition to 14-day anaerobic, fungal, and acid-fast bacilli (AFB) cultures. Additionally, right prepatellar bursa tissue cultures were sent. Gram stain was positive for gram-positive rods. Postoperatively, he was continued on IV vancomycin. A positive abscess gram stain result necessitated an infectious disease (ID) consultation. The ID team recommended following final cultures and continuing empiric IV vancomycin. The patient did not oblige to staying inpatient as recommended by the ID team for culture-guided definite therapy but was willing to be followed up in the outpatient clinic. He discharged against medical advice (AMA); before discharge, he received a single IV dalbavancin 1.5 gm dose. An orthopedic, IM, and ID follow-up was arranged before discharge. Blood cultures, AFB cultures, and fungal cultures returned negative. The abscess fluid and the prepatellar tissue cultures both grew *C. ulcerans*. The susceptibility pattern indicated that it was intermediate to penicillin and sensitive to ceftriaxone, meropenem, and vancomycin (Table 1). At a two-week follow-up, the patient's pain had improved with decreased warmth and swelling. Clinical examination revealed superficial dehiscence and mild tenderness on palpation. He received another two weeks of oral cefdinir to complete therapy. At a six-week follow-up with the orthopedics, the incision site had healed with joint mobility and weight-bearing recovery.

## 3. Discussion

*C. ulcerans* is a nonlipophilic, fermentative *Corynebacterium* subspecies belonging to the *C. diphtheriae* group, which causes mastitis in cattle and airway disease in other livestock [1]. Its reservoir includes livestock, pet animals, squirrels, otters, camels, monkeys, and other wild animals [3]. It can produce the diphtheria toxin, which is 95% homologous to the toxin produced by *C. diphtheriae* [3]. Potential virulence factors are the diphtheria-like toxin (DT), phospholipase D, Shiga-like ribosome binding protein, endoglycosidase of the EndoE family, sialidase, adherence pili and complex cell wall containing peptidoglycan, and an outer mycolic acid layer [3]. Phospholipase D and DT can cause local tissue necrosis [4]. An experimental in vivo mouse model indicates that the high virulence of *C. ulcerans* is independent of DT production [3]. DT is a proteinaceous exotoxin with tropism for the myocardium, nervous system, kidneys, and suprarenal glands. Zoonotic transmission results in airway disease, skin and soft tissue infections, and tonsil, peritoneum, lymph node, and ear infections. Transmission occurs via raw milk consumption and contact with animal wounds or secretions. Transmission from wild animals to livestock has been suggested [3]. The incubation period is one to six days [4]. An increase in *C. ulcerans* zoonosis was observed in industrialized nations recently [4]. The prevalent risk factor has been animal contact in most cases. The zoonotic transmission has been verified in multiple cases, whereas human-to-human transmission has been proven in a single case [3].

Clinical manifestations are variable based on the infection site. Respiratory illness similar to diphtheria is the most common manifestation, and in some countries, it is more frequent than *C. diphtheria* [5]. Upper airway disease presents with a sore throat which can progress to a swollen bull neck with clinical examination revealing a firmly adherent pseudomembrane. If unattended, this can cause airway obstruction. Lower airway disease is rare in comparison. The next most common presentation is the cutaneous disease, which manifests as an erythematous tender rolled edge ulcer with adjacent edema. DT can also cause myocarditis, peripheral neuropathy, and bulbar dysfunction. It is seen commonly with respiratory illness and is rare in extensive cutaneous disease [5]. Rare clinical presentations include peritonitis, caseous lymphadenitis, and bronchopneumonia [3, 7, 8].

Diagnosis is arrived at by reviewing symptomatology, physical examination, and microbiologic evaluation. Clinical specimens include oropharyngeal and nasal mucosa, cutaneous lesions of both patients, and suspected animal contacts for the prior two weeks. Bacterial growth media selected are blood agar and tellurite-containing agar [3]. Species-level identification is favored if cultured from sterile sites (blood or cerebrospinal fluid) or is the prevalent organism on gram stain or urine culture [1]. Vitek or matrix-assisted laser desorption ionization-time of flight mass spectrometry is used to identify the bacteria precisely. A modified Elek test or a polymerase chain-based genotypic test can detect toxin production [3]. A matrix-assisted laser desorption ionization-time of flight mass spectrometry (MALDI-TOF MS) review reveals exceptional results in comparison to conventional methods and helps in identifying accurately up to genus and species level (>90%) [9]. A MALDI-TOF MS-based instrument database needs to be updated when used to detect the *Corynebacterium* microorganism. Based on Clinical and Laboratory Standards Institute breakpoints, 76% of *C. ulcerans* are intermediate to penicillin, 97% are susceptible to erythromycin, and 100% are susceptible to vancomycin. Only 3% of *Corynebacterium* species are resistant to erythromycin [10]. Dalbavancin is as efficacious as vancomycin against *Corynebacterium* species [11, 12]. Erythromycin is the drug of choice for *C. ulcerans* infections [10]. For severe disease, diphtheria antitoxin (DAT) at the earliest is recommended as it inhibits the systematic toxin effects [5]. Antimicrobial susceptibility can detect resistance patterns to modify, deescalate, or use an alternative agent for therapy. Antibodies against diphtheria toxoid decline by 10% annually, so decennial vaccination is needed [4]. Diphtheria toxoid possibly decreases disease severity with no effect on colonization and infection [2, 4]. Most patients with *C. ulcerans* infections have partial or complete diphtheria toxoid vaccination and are above 60 yrs [4]. Droplet and contact precautions should be followed as in diphtheria [13]. Disease elimination will require immediate antibiotic therapy, decreasing symptomatic carrier transmission risk, and decennial vaccination in adults.

Our patient had multiple sources of infection, including hogs and pet animals. He has recurrent minor abrasions over the knee during his farm work. He likely suffered less severe disease due to his full diphtheroid toxoid vaccination status. Our institution lacked laboratory capability to identify toxin production. Appropriate antimicrobial therapy and surgical intervention resulted in an excellent recovery. Physicians should use the guidance provided for diphtheria treatment for *C. ulcerans* infections. As *C. ulcerans* is not a notifiable disease, no surveillance measures were available to identify the transmission source. A PubMed literature review reveals no prior cases. The toxigenicity of isolated cultures should be assessed. *C. ulcerans* disease's infrequent occurrence and the lack of specimen culture sampling result in delayed diagnosis with grave implications.

## 4. Conclusion

This is the first case of prepatellar bursitis with abscess due to *C. ulcerans*. *C. ulcerans* needs to be considered in the correct epidemiological setting, particularly where zoonotic transmission is suspected. Physicians must be cognizant of disease acquisition from pet animals and its pathogenicity. Only a few cases have been described before in the USA. It is not a notifiable disease, as per the Centers for Disease Control and Prevention. For toxigenicity and species confirmation, it is ideal to send the isolate to a reference laboratory. Animal reservoirs can perpetuate infection in the community. In the future, the incidence may increase, leading to more diverse disease manifestations. Disease monitoring is essential to detect and prevent its spread. A recommendation for close contacts is vaccination. DAT therapy should be considered in patients with severe disease. Diphtheria vaccine decennial boosters in adults may provide some immunity against this infection, especially against toxin production, and decrease clinical severity. Current clinical laboratories in the USA cannot detect toxins due to a lack of expertise. The presence of *Corynebacteria* in clinical specimens should be processed judiciously.

## Figures and Tables

**Table 1:** Antibiotic susceptibility.

Organism	*Corynebacterium ulcerans*	
Antibiotic	MIC (mcg/mL)	Interpretation
Penicillin	1	Intermediate
Ceftriaxone	1	Susceptible
Meropenem	≤0.25	Susceptible
Vancomycin	≤1	Susceptible

MIC = minimum inhibitory concentration.

## References

[1] Kim R., Reboli A. C. (2020). *Mandell, Douglas, and Bennett's Principles and Practice of Infectious Diseases*.

[2] Otshudiema J. O., Acosta A. M., Cassiday P. K., Hadler S. C., Hariri S., Tiwari T. S. P. (2020). Respiratory illness caused by Corynebacterium diphtheriae and C. ulcerans, and use of diphtheria anti-toxin in the United States, 1996-2018. *Clinical Infectious Diseases*.

[3] Hacker E., Antunes C. A., Mattos-Guaraldi A. L., Burkovski A., Tauch A. (2016). Corynebacterium ulcerans, an emerging human pathogen. *Future Microbiology*.

[4] Dias A. A., Santos L. S., Sabbadini P. S. (2011). Difteria pelo Corynebacterium ulcerans: uma zoonose emergente no Brasil e no mundo. *Revista de Saúde Pública*.

[5] Moore L. S. P., Leslie A., Meltzer M., Sandison A., Efstratiou A., Sriskandan S. (2015). *Corynebacterium ulcerans* cutaneous diphtheria. *The Lancet Infectious Diseases*.

[6] Weil L. M., Butler C., Howell K. R. (2019). Notes from the field: conjunctivitis caused by toxigenic Corynebacterium ulcerans - Missouri, 2018. *MMWR. Morbidity and Mortality Weekly Report*.

[7] Kimura Y., Watanabe Y., Suga N. (2011). Acute peritonitis due to Corynebacterium ulcerans in a patient receiving continuous ambulatory peritoneal dialysis: a case report and literature review. *Clinical and Experimental Nephrology*.

[8] Yasuda I., Matsuyama H., Ishifuji T. (2018). Severe pneumonia caused by toxigenic Corynebacterium ulcerans infection, Japan. *Emerging Infectious Diseases*.

[9] Zasada A. A., Mosiej E. (2018). Contemporary microbiology and identification of Corynebacteria spp. causing infections in human. *Letters in Applied Microbiology*.

[10] Marosevic D. V., Berger A., Kahlmeter G., Payer S. K., Hörmansdorfer S., Sing A. (2020). Antimicrobial susceptibility of Corynebacterium diphtheriae and Corynebacterium ulcerans in Germany 2011-17. *The Journal of Antimicrobial Chemotherapy*.

[11] Goldstein E. J., Citron D. M., Merriam C. V., Warren Y., Tyrrell K., Fernandez H. T. (2003). In vitro activities of dalbavancin and nine comparator agents against anaerobic gram-positive species and corynebacteria. *Antimicrobial Agents and Chemotherapy*.

[12] Gales A. C., Sader H. S., Jones R. N. (2005). Antimicrobial activity of dalbavancin tested against Gram-positive clinical isolates from Latin American medical centres. *Clinical Microbiology and Infection*.

[13] Lartigue M. F., Monnet X., le Flèche A. (2005). Corynebacterium ulcerans in an immunocompromised patient with diphtheria and her dog. *Journal of Clinical Microbiology*.

